# Real-world effectiveness of total flavonoids from *Desmodium styracifolium* for ureteral stones: a propensity score-matched retrospective cohort study

**DOI:** 10.3389/fphar.2026.1829360

**Published:** 2026-07-27

**Authors:** Zhongbao Zhou, Baining Zhang, Zhuoqi Cheng, Yong Zhang, Yumeng Chai

**Affiliations:** 1 Department of Urology, Beijing Tiantan Hospital, Capital Medical University, Beijing, China; 2 Department of Internal Medicine 3, Rheumatology and Immunology, Friedrich-Alexander-Universität Erlangen-Nürnberg (FAU) and Universitätsklinikum Erlangen, Erlangen, Germany

**Keywords:** Desmodium styracifolium, flavonoids, medical expulsive therapy, phytotherapy, ureteral calculi

## Abstract

**Background:**

Ureteral stones frequently lead to urinary tract obstruction, and medical expulsive therapy (MET) continues to serve as a mainstay of non-invasive management. Total flavonoids from *Desmodium styracifolium* (TFDS), a traditional Chinese herbal extract, have been suggested to promote stone passage, but real-world evidence is limited.

**Methods:**

We performed a retrospective cohort study including 341 outpatients diagnosed with unilateral ureteral stones between January and December 2024. Patients receiving TFDS were classified as the study group (n = 194), while those not receiving TFDS served as controls (n = 147). We used 1:1 propensity score matching to ensure comparable baseline profiles, yielding 142 matched pairs. The primary outcomes were stone expulsion time and expulsion rate within 30 days. Subgroup analyses were performed according to stone size and location.

**Results:**

After PSM, all baseline variables were well balanced between the two groups. Patients treated with TFDS experienced a shorter average duration to stone passage than controls (9.06 vs. 12.24 days, p = 0.003). Subgroup analyses showed that TFDS shortened expulsion time in both small (<5 mm) and larger (five to nine mm) stones, as well as across different ureteral locations, with the most marked benefit observed in upper ureteral stones. The overall stone expulsion rate was higher in the TFDS group than in controls (63.4% vs. 47.9%, p = 0.09), with significant improvement in stones 5–9 mm (p = 0.021) and in middle-to-lower ureteral stones (p = 0.011). No adverse drug reactions were reported.

**Conclusion:**

After adjusting for baseline differences using PSM, TFDS significantly shortened stone expulsion time and improved expulsion rates, particularly for larger and distally located ureteral stones. TFDS appears to be a safe and effective adjunct to MET in the conservative management of ureteral stones.

## Introduction

Ureteral calculi are a prevalent manifestation of urinary stone disease, which affects an estimated 11% of the global population. In 2021, over 106 million incident cases were reported worldwide, representing a 26.7% increase since 2000, with a concomitant 34.5% rise in disability-adjusted life years (DALYs) attributable to urolithiasis (Zyoud et al., 2024; GBD, 2021 Urol ithiasis Collaborators, 2024). Despite the availability of ureteroscopy and extracorporeal shock wave lithotripsy (ESWL), conservative measures including watchful waiting, aggressive hydration (≥2.5 L/day), analgesia with nonsteroidal anti-inflammatory drugs (NSAIDs), and medical expulsive therapy (MET) with α-adrenergic blockers or calcium channel blockers still represent the first-line choice for selected cases (De Coninck et al., 2019). Stone expulsion probability is influenced by factors such as stone size, anatomical location, ureteral peristalsis, local edema, and smooth muscle spasm. Stones <6 mm has high likelihood of spontaneous passage (Türk et al., 2016a). As such, pharmacologic strategies to facilitate stone passage have gained increasing attention in clinical urology (Monti et al., 2016).

Among the herbal preparations traditionally used in China, *Desmodium styracifolium*, commonly referred to as “Guang Jin Qian Cao”, has been historically employed for its diuretic, anti-inflammatory, and litholytic properties (Opryshko et al., 2024). Extracts derived from this plant, particularly total flavonoids, have been shown in both preclinical and clinical studies to exert beneficial effects on urinary stone disease (Zhou et al., 2018). In a rat model of calcium oxalate urolithiasis, total flavonoids from *D. styracifolium* (TFDS) significantly reduced renal crystal deposition and urinary calcium excretion while upregulating osteopontin expression, a key inhibitor of crystal adhesion (Zhou et al., 2018). *In vitro*, the phenolic hydroxyl and carbonyl groups of flavonoids have been shown to chelate calcium ions, thereby inhibiting calcium oxalate crystal nucleation, growth, and aggregation (Xiang et al., 2015; Miyaoka and Monga, 2009). Clinically, a multicenter randomized controlled trial (RCT) demonstrated that TFDS capsule treatment achieved a significantly higher stone expulsion rate compared with placebo in patients with 5–10 mm ureteral stones (52.1% vs. 22.2%, p = 0.029), with a favorable safety profile (Liu et al., 2023). Another recent study reported that adjuvant TFDS therapy following flexible ureteroscopic lithotripsy improved the stone-free rate from 88.9% to 98.8% without increasing adverse events (Gao et al., 2024). This evidence supported the role of TFDS as a potential adjunct in the non-invasive management of urinary calculi, though large-scale real-world studies remain limited.

In this real-world retrospective cohort study, we aim to evaluate the effectiveness of TFDS as an adjunct to MET for ureteral stones. By leveraging electronic medical records (EMR) and applying propensity score matching (PSM) to minimize baseline confounding, we seek to provide robust evidence for the integration of traditional phytotherapy into modern urological practice and to inform strategies for optimizing conservative management of uncomplicated ureteral calculi.

## Methods

### Study design

Ethical approval for this retrospective cohort investigation was obtained from Beijing Tiantan Hospital (KY 2025-326–01). Given the retrospective design, the requirement for written informed consent was waived. All patient information was completely anonymized before analysis to maintain confidentiality. The study adhered to the principles outlined in the Declaration of Helsinki. Patients diagnosed with unilateral ureteral stones between January and December 2024 were included. This study was conducted and reported in accordance with the Strengthening the Reporting of Observational Studies in Epidemiology (STROBE) guidelines for observational studies (Von et al., 2007). The checklist was used to ensure that all essential items were appropriately addressed.

Patients diagnosed with unilateral ureteral stones between January and December 2024 at the urology outpatient clinic of Beijing Tiantan Hospital were included. The study population was divided into two groups: a study group that received TFDS capsules in addition to standard conservative management, and a control group that received standard conservative management alone. PSM at a 1:1 ratio was utilized to minimize baseline differences and balance covariates between the groups. The PSM procedure accounted for age, gender, stone size, location of the stone, and laterality.

### Intervention

Patients in the study group received TFDS capsules (Guangjinqiancao Zonghuangtong Jiaonang; Wuhan Guanggu Renfu Biotechnology Co., Ltd., NMPA approval number: GuoYaoZhunZi Z20220,002), administered orally at a dose of 0.6 g (three 0.2 g capsules, each containing 133 mg of total flavonoid extract) three times daily. Treatment was continued for 28 days or until spontaneous stone expulsion was confirmed.

Patients in the control group did not receive TFDS but were managed according to the standard conservative protocol, which consisted of: (i) aggressive oral hydration (≥2.5 L of water per day), (ii) analgesia with NSAIDs as needed for renal colic, and (iii) watchful waiting with scheduled follow-up. Both groups received identical instructions regarding hydration targets, physical activity (encouragement of ambulation), and urine straining to capture passed stones. Concomitant use of other MET agents (e.g., α-adrenergic blockers, calcium channel blockers) was not systematically documented in the EMR; this is addressed as a study limitation.

### Inclusion and exclusion criteria

Inclusion required that participants met all of the following conditions: (1) Aged between 18 and 80 years; (2) Diagnosed with a single ureteral stone located below the pelvi-ureteric junction (UPJ) with a maximum diameter of ≤10 mm on non-contrast CT or ultrasound; (3) Normal urinary tract anatomy confirmed by imaging; (4) No evidence of urinary tract infection or systemic inflammatory response, such as high fever or clinical signs of urosepsis. Exclusion applied to participants meeting any of the following conditions: (1) Known allergy or hypersensitivity to *Desmodium styracifolium* or its active components, including total flavonoids; (2) Severe hydronephrosis confirmed by imaging; (3) Pregnant or lactating women; (4) Multiple ureteral stones or bilateral ureteral stones; (5) Prior urological intervention (ureteroscopy, ESWL, or percutaneous nephrolithotomy) for the index stone episode.

## Data collection and variables

All clinical data were extracted from the EMR system using a standardized data collection form. The following variables were recorded for each patient: age, sex, treatment group assignment (TFDS vs. control), date of initial visit, and date of confirmed stone expulsion or surgical intervention.

Imaging data were abstracted from the official radiology reports of non-contrast CT, ultrasonography, or kidney-ureter-bladder radiography (KUB) performed at the index visit. Specifically, the following parameters were extracted: stone maximum diameter (mm), stone laterality (left/right), stone location (upper/middle/lower ureter, with the upper border of the sacroiliac joint as the anatomical landmark demarcating upper from middle ureter), presence and grade of hydronephrosis and presence of renal stones. When multiple imaging modalities were available for the same patient, CT measurements were prioritized, followed by ultrasound data.

Given the retrospective study design and reliance on EMR data, information on concurrent medications, specifically α-adrenergic blockers (e.g., tamsulosin), NSAIDs, calcium channel blockers, and other MET-related agents, was not systematically available for all patients and therefore could not be included as covariates in the analysis. This limitation is further addressed in the discussion. All data were anonymized prior to analysis, and patient confidentiality was maintained throughout the study in accordance with institutional and ethical guidelines. All patients were followed for 30 days. Follow-up assessments included patient-reported stone passage, symptom evaluation (pain, hematuria), urinalysis, and imaging with ultrasonography or CT as needed.

### Outcome measures

The primary outcome was stone expulsion time, defined as the number of days from the initial clinic visit to the date of confirmed spontaneous stone passage. Stone passage was confirmed by one or more of the following: patient report of visualized stone passage with urine straining, absence of the stone on follow-up imaging (ultrasonography or CT), or resolution of hydronephrosis on imaging in conjunction with symptom relief. The secondary outcome was stone expulsion rate within 30 days, defined as the proportion of patients who achieved spontaneous stone passage within 30 days of the index visit. Patients who underwent surgical intervention (ureteroscopy or ESWL) before day 30 or who were lost to follow-up were classified as not achieving spontaneous stone expulsion.

### Subgroup definitions and follow-up

Prespecified subgroup analyses were conducted according to two clinical strata: (1): stone size, dichotomized as <5 mm and 5–9 mm; and (2) stone location, categorized as upper ureteral (from the UPJ to the upper border of the sacroiliac joint) and middle-to-lower ureteral (from the upper border of the sacroiliac joint to the ureterovesical junction). Middle and lower ureteral stones were pooled for analysis because of the small number of isolated middle ureteral stones and their similar physiological behavior regarding spontaneous passage.

### Statistical analysis and power considerations

Descriptive statistics were employed to summarize patient characteristics, with continuous variables reported as mean ± standard deviation (SD), and categorical variables as counts and percentages. Prior to PSM, differences between the study and control groups were evaluated using the Mann–Whitney U test for continuous variables (such as age and stone size) and the chi-square test for categorical variables (including gender and laterality). For analyses following PSM, the Mann–Whitney U test was employed to compare expulsion times, and expulsion rates were examined using McNemar’s or paired chi-square tests. Subgroup analyses by stone size and location were performed similarly. All statistical tests were two-sided, with p < 0.05 indicating significance. Analyses were carried out using SPSS version 23.0 (IBM Corp., Armonk, NY, USA).

As this was a retrospective cohort study, the sample size was determined by the number of eligible patients identified during the study period (January-December 2024) rather than by an *a priori* power calculation. To evaluate whether the obtained sample was sufficient to detect clinically meaningful differences, a *post hoc* power analysis was performed. For the primary endpoint (stone expulsion time), with 142 patients per group and the observed mean difference of 3.18 days (pooled SD ≈ 5.78 days, Cohen’s d ≈ 0.55), the study had approximately 87% power at a two-sided α of 0.05, indicating adequate statistical power for the primary analysis. For the secondary endpoint (stone expulsion rate within 30 days), with observed rates of 63.4% vs. 47.9% (absolute difference 15.5 percentage points), the achieved power was approximately 62%, suggesting that the study was underpowered to detect this magnitude of difference in the binary outcome. The borderline p-value of 0.09 for this endpoint may therefore reflect a Type II error rather than a true absence of treatment effect, and the clinically meaningful 15.5-percentage-point absolute difference should be interpreted with this consideration in mind.

## Results

### Baseline characteristics of the study population

A total of 284 patients were retained for analysis after PSM, including 142 in the TFDS group and 142 in the control group. The baseline demographic and clinical characteristics of the two groups before and after PSM are summarized in Table 1.

**TABLE 1:** Baseline characteristics of patients before and after PSM.

​	Before PSM			After PSM		
Group	Study group (n = 194)	Control group (n = 147)	P Value	Study group (n = 142)	Control group (n = 142)	P Value
Age, years (mean ± SD)	44.2 ± 14.2	46.1 ± 14.1	0.189	44.5 ± 14.45	45.6 ± 14.0	0.413
Gender, n (%)						
Male	153 (78.9)	107 (72.8)	0.192	107 (75.4)	107 (75.4)	1
Female	41 (21.1)	40 (27.2)		35 (24.6)	35 (24.6)	
Stone size, mm (mean ± SD)	5.6 ± 2.0	5.7 ± 1.8	0.283	5.7 ± 2.0	5.7 ± 1.7	0.845
Stone size						
<5 mm	67 (34.5)	43 (29.3)	0.301	47 (33.1)	43 (30.3)	0.61
5–9 mm	127 (65.5)	104 (70.7)		95 (66.9)	99 (69.7)	
Laterality						
Left, n (%)	100 (51.5)	85 (57.8)	0.249	80 (56.3)	80 (56.3)	1
Right, n (%)	94 (48.5)	62 (42.2)		62 (43.7)	62 (43.7)	
Stone location						
Upper ureteral, n (%)	97 (50.0)	78 (53.1)	0.894	76 (53.5)	77 (54.2)	0.895
Middle ureteral, n (%)	39 (20.1)	21 (14.3)		24 (16.9)	19 (13.4)	
Lower ureteral, n (%)	58 (29.9)	48 (32.7)		42 (29.6)	46 (32.4)	

Study group treated with TFDS; SD: standard deviation; PSM: propensity score matching.

The study and control groups were comparable with respect to age (44.5 ± 14.5 vs. 45.6 ± 14.0 years, p = 0.413), gender distribution (male: 75.4% vs. 75.4%, p = 1.000), stone laterality (left side: 56.3% vs. 56.3%, p = 1.000), or mean stone size (5.7 ± 2.0 mm vs. 5.7 ± 1.7 mm, p = 0.845).

When categorized by stone size, the proportion of patients with stones <5 mm (33.1% vs. 30.3%) and 5–9 mm (66.9% vs. 69.7%) did not vary significantly between the study and control groups (p = 0.610). Similarly, stone location was well balanced after matching (p = 0.895), with upper ureteral stones accounting for 53.5% in the TFDS group and 54.2% in the control group, middle ureteral stones for 16.9% and 13.4%, and lower ureteral stones for 29.6% and 32.4%, respectively.

These results indicate that the matching procedure effectively mitigated baseline discrepancies between the groups, ensuring comparability for subsequent outcome analyses.

## Stone expulsion outcomes

After propensity score matching, a total of 284 patients, with 142 in each group, were included in the outcome analysis. The overall stone expulsion time was significantly shorter in the TFDS-treated study group compared with the control cohort (9.06 vs. 12.24 days, p = 0.003, Table 2), suggesting that treatment with TFDS promoted faster stone passage.

**TABLE 2:** Comparison of stone expulsion outcomes between the study and control groups after PSM.

Outcomes	Study group (n = 142)	Control group (n = 142)	P Value
Stone expulsion time			
Overall, (days)	9.06	12.24	0.003
Stone size subgroup			
<5 mm	8.06	11.85	0.023
5–9 mm	9.6	12.97	0.039
Stone location subgroup			
Upper ureter	9.62	14.41	0.022
Middle and lower ureter	8.68	10.62	0.029
Stone expulsion rate within 30 days			
Overall, n (%)	90/142 (63.38)	68/142 (47.89)	0.09
Stone size subgroup			
<5 mm	34/47 (72.34)	26/43 (60.47)	0.233
5∼9 mm	56/95 (58.95)	42/99 (42.42)	0.021
Stone location subgroup			
Upper ureter	37/76 (48.68)	29/77 (37.66)	0.169
Middle and lower ureter	53/66 (80.3)	39/65 (60)	0.011

The secondary endpoint (stone expulsion rate within 30 days) was achieved in 90 of 142 patients (63.4%) in the TFDS group and 68 of 142 patients (47.9%) in the control group. Although the TFDS group showed a numerically higher expulsion rate, this overall difference did not reach statistical significance (p = 0.09; Table 2).

### Subgroup analyses

*By stone size*: When stratified by stone size, patients with stones <5 mm showed a significantly shorter mean expulsion time in the TFDS group compared with controls (8.06 vs. 11.85 days, p = 0.023; Table 2). Similarly, for stones measuring 5–9 mm, the TFDS group demonstrated a shorter expulsion time (9.60 vs. 12.97 days, p = 0.039; Table 2). Regarding expulsion rate, a significant improvement was observed in the TFDS group for stones 5–9 mm (58.9% vs. 42.4%, p = 0.021; Table 2), while no significant difference was found for stones <5 mm (72.3% vs. 60.5%, p = 0.233; Table 2).

*By stone location*: The TFDS group exhibited significantly shorter expulsion times for both upper ureteral stones (9.62 vs. 14.41 days, p = 0.022; Table 2) and middle-to-lower ureteral stones (8.68 vs. 10.62 days, p = 0.029; Table 2). The expulsion rate was significantly higher in the TFDS group for middle-to-lower ureteral stones (80.3% vs. 60.0%, p = 0.011; Table 2), whereas no significant difference was observed for upper ureteral stones (48.7% vs. 37.7%, p = 0.169; Table 2).

These results indicate that TFDS may effectively accelerate ureteral stone expulsion and increase the likelihood of spontaneous passage, with the most consistent benefit observed for stones 5–9 mm in size and those located in the middle-to-lower ureter.

## Discussion

This retrospective, PSM cohort study evaluated the clinical effectiveness of TFDS in promoting spontaneous ureteral stone expulsion. After matching, patients receiving TFDS exhibited significantly shorter mean stone expulsion time compared with the control group, with the greatest benefit observed for stones measuring 5–9 mm and those located in the middle or upper ureter. The overall expulsion rate was also higher among TFDS users, particularly for middle and lower ureteral stones.

No adverse drug reactions attributable to TFDS were documented in the EMR during the 30-day follow-up period. However, given the retrospective design, this finding must be interpreted with caution. Unlike prospective clinical trials, which use systematic adverse event monitoring and standardized severity grading, retrospective studies rely on spontaneous documentation by treating clinicians in routine practice. Mild or non-specific symptoms may not have been recorded, and the absence of documented events cannot be equated with the absence of actual events. The favorable safety profile observed in prior prospective studies of TFDS provides supportive evidence (EAU Guidelines Office, 2025), but definitive safety conclusions require dedicated prospective pharmacovigilance.

Our findings align well with those of Liu et al., who conducted a randomized, double-blind, placebo-controlled trial across multiple centers on TFDS capsules in 66 patients with ureteral stones (0.5–1.0 cm). A markedly higher stone expulsion rate was reported in patients receiving TFDS compared with those given placebo (52.1% vs. 22.2%, p = 0.029), with good safety and tolerability (Liu et al., 2023). Similarly, Gao et al. (2024) demonstrated that TFDS could effectively promote the clearance of residual stones following flexible ureteroscopic lithotripsy, improving the stone-free rate from 88.9% to 98.8% without increasing adverse events, while also showing favorable cost-effectiveness (Gao et al., 2024). These clinical data, together with our results, provide converging evidence supporting TFDS as a safe and effective adjunctive agent in the management of urinary calculi, both for spontaneous passage and postoperative clearance (Sorokin et al., 2017).

MET remains a widely accepted first-line strategy in the management of uncomplicated ureteral stones, especially those under 10 mm in diameter (Türk et al., 2016b). While α-blockers, calcium channel blockers, and NSAIDs have been extensively studied for MET, interest has grown in the use of phytotherapeutics as complementary or alternative agents (Campschroer et al., 2018; Morsy et al., 2022). Several traditional Chinese medicines (TCMs) have demonstrated promising effects in facilitating ureteral stone expulsion and preventing recurrence. Shilintong (Herba Lysimachiae), one of the most commonly used herbs, has been reported to exert diuretic, anti-inflammatory, and smooth muscle-relaxing effects that promote stone passage and reduce urinary tract irritation (Lai et al., 2018). Clinical studies have shown that Shilintong extracts can shorten stone expulsion time and increase stone-free rates when used alone or in combination with α-blockers (Kai et al., 2025; Dahai and Zongzhi, 2014). Wulingsan, a classical multi-herb formula containing Poria, Alisma, and Polyporus, has also been investigated for its role in stone prevention. Several studies have shown that Wulingsan was effective in the treatment of urinary calculi, reducing short-term recurrence rates and improving stone expulsion rates (Yuan, 2016; Lin et al., 2013). Mechanistically, Wulingsan enhances urinary citrate excretion and inhibits calcium oxalate crystal aggregation (Junbiao et al., 2013). Several other TCM formulas, such as Jineijin, and Bazhengsan, have been reported to aid in stone clearance through diuretic, anti-inflammatory, and smooth muscle-relaxing effects (Yan et al., 2002; Deke, 2025). However, most of these agents lack large-scale clinical trials or rigorous mechanistic studies to support their efficacy.

Compared with these agents, TFDS exhibits a more clearly defined chemical composition, primarily composed of total flavonoids. *Desmodium styracifolium*, a traditional Chinese medicinal herb, has long been used to treat urolithiasis (Xiang et al., 2015). Research indicated that flavonoid compounds, particularly the phenolic hydroxyl and carbonyl groups in *D. styracifolium*, played a pivotal role in stone dissolution (Wang et al., 2024). These compounds can interact with calcium ions in the urine, reducing the concentration of calcium crystals and preventing their further aggregation into larger stones (Miyaoka and Monga, 2009). This interaction not only reduces the propensity for urinary stone formation but also promotes the breakdown of pre-existing calcium oxalate stones. In addition to its role in inhibiting crystal growth, *D. styracifolium* has shown significant anti-inflammatory properties (Opryshko et al., 2024). Studies have demonstrated that its flavonoids can reduce local inflammation in the urinary tract, a manifestation commonly seen in patients with urolithiasis (Liu et al., 2013). Furthermore, *D. styracifolium* has a mild diuretic effect, increasing urine output, which further helps in flushing out the stone fragments and reducing urinary retention (Opryshko et al., 2024).

Subgroup evaluation demonstrated that TFDS had the greatest efficacy in the middle and lower ureter, where it not only reduced expulsion time but also increased passage rates. This may be due to improved ureteral smooth muscle coordination and enhanced peristaltic activity induced by the flavonoid compounds. Although the upper ureter subgroup also showed a significant reduction in expulsion time, the increase in passage rate did not reach statistical significance, possibly because of anatomical resistance near the ureteropelvic junction and a smaller sample size in this subgroup.

Importantly, no adverse events were observed during TFDS administration in our study, consistent with prior clinical trials. Compared with α-blockers and calcium channel blockers, standard agents in MET, TFDS offers an advantage of minimal systemic side effects such as hypotension, dizziness, or gastrointestinal discomfort (Campschroer et al., 2018). This favorable safety profile suggests that TFDS can be considered a suitable and well-tolerated option, especially for patients with contraindications to traditional MET drugs or those seeking phytotherapeutic alternatives.

Notwithstanding the study’s strengths, several potential limitations exist. Firstly, the retrospective design may have introduced selection bias, although baseline characteristics were well balanced between groups. Second, stone expulsion was primarily assessed through patient self-report and ultrasound follow-up, which may have led to under- or over-estimation of actual expulsion time. Third, long-term recurrence and stone composition were not evaluated, which could have further clarified the mechanism of action. Fourth, patients with small concomitant renal stones (<5 mm) were not excluded from the analysis, as documented in our data collection record of “presence of renal stones.” While we did not exclude such patients, the clinical impact of small asymptomatic renal calculi on ureteral stone passage outcomes is expected to be minimal. Fifth, due to the retrospective design and reliance on EMR data, information on concurrent medications, including α-adrenergic blockers, NSAIDs, and calcium channel blockers, was not systematically recorded for all patients. As a result, we were unable to adjust for or stratify by the use of standard MET agents, which may have contributed to residual confounding. Sixth, the prespecified subgroup analyses were conducted without formal adjustment for multiplicity. Given the relatively small sample sizes within each stratum, the probability of false-positive findings is elevated. Seventh, several potentially confounders could not be captured from the EMR and were therefore not included in the PSM or adjusted analyses. These include factors known to influence stone formation and spontaneous passage, such as body mass index (BMI), metabolic stone risk profile, stone composition, serum uric acid level, smoking status, dietary habits, and daily fluid intake volume. Future prospective studies should incorporate standardized documentation of all concomitant medications to enable more precise estimation of the independent treatment effect of TFDS.

In conclusion, TFDS appear to be an effective and safe option to enhance spontaneous ureteral stone expulsion, particularly in patients with larger stones or stones located in the upper and mid ureter. This phytotherapeutic agent may serve as a valuable adjunct in the non-invasive management of urolithiasis, offering both efficacy and tolerability in real-world outpatient settings.

## Data Availability

The original contributions presented in the study are included in the article/supplementary material, further inquiries can be directed to the corresponding authors.
